# Development of Telenursing Guidelines to Improve the Quality of Services in Diabetic Wound Care in a Hospital in Thailand: Case Study

**DOI:** 10.2196/74228

**Published:** 2026-02-09

**Authors:** Chonlada Darayon, Paralee Opasanant, Siriporn Sangsrijan, Waraporn Pattaramungkhunkat, Panpimol Sukwong

**Affiliations:** 1 Nursing Department, Mae Chan Hospital, Mae Chan, Chiang Rai, Thailand; 2 School of Nursing, University of Phayao, 19 Moo 2, Tambon Maeka, Amphur Muang, Phayao, 5600, Thailand, 66 0835805179; 3 School of Nursing, Northern College, Tak, Tak, Thailand

**Keywords:** telenursing, diabetic ulcers, wound care, nursing guideline, quality of service

## Abstract

**Background:**

The majority of patients with diabetic wounds living in Mae Chan District, Chiang Rai Province face challenges such as a shortage of nurses, limited access to health care, and insufficient resources. Strategies such as specialist networks, patient monitoring, and online care platforms are crucial to improving diabetic wound management in the community.

**Objective:**

This study aims to develop telenursing guidelines for caring for patients with diabetic wounds and foot ulcers, and to investigate the effects of telenursing on wound healing among patients.

**Methods:**

Participatory action research was conducted in three cycles: (1) assessing the current situation and feasibility of telenursing; (2) evaluating telenursing guidelines for wound healing; and (3) examining the effects of telenursing on wound healing, amputation rates, and patient satisfaction.

**Results:**

The mean diabetic wound severity scores decreased after receiving telenursing care at weeks 2, 4, 6, and 8 (*P*<.001). No patients were found to have foot or leg amputations. The patients in the group who received telenursing care showed that their wounds healed in an average of 8.6 (SD 4.3) weeks. The satisfaction score for telenursing care was 4.7 out of 5 (SD 0.2).

**Conclusions:**

Telenursing guidelines were developed to enhance access to wound care, reduce amputation rates, and promote wound healing, resulting in a significant reduction in wound severity and the absence of amputations. The study further demonstrated that telenursing not only expedited healing times but also reduced health care costs and improved patient satisfaction.

## Introduction

Diabetes is one of the serious chronic diseases strongly associated with health complications, including macrovascular disease, microvascular disease, peripheral vascular complications, and neuropathy, leading to serious health problems such as coronary heart disease, stroke, kidney disease, and retinal abnormalities. In addition, patients with diabetes are at risk of having foot ulcers, which are the most common complications causing disability, amputation, or premature death. This is one of the most critical public health problems. Diabetic foot ulcers are among the most common complications in persons with diabetes who have problems with uncontrolled blood sugar [1]. The incidence shows that patients with diabetes develop foot ulcers at 19% to 34% and have recurrence symptoms within 3 to 5 years at 65%; moreover, foot or leg amputation is found to be shared among patients with diabetes compared to the general population at 50% to 70%, and they have a chance of premature death within 5 years [2]. Foot ulcers among persons with diabetes take longer to recover than those of normal people, taking an average of 11 to 14 weeks. This could affect the lifestyles of the patients because it causes physical, mental, and social limitations. According to a study of health expenses for caring for patients with foot ulcers in a tertiary university hospital in Singapore between 2013 and 2017, the average cost of wound care alone was US $3368 per patient per year; the cost of minor amputation below the ankle was US $10,468 per patient per year; and the cost of major amputation above or below the knee was US $30,131 per patient per year [3].

Foot ulcers [4,5] can be classified into the following types of wounds. First, infected ulcers are where the wound is infected and becomes inflamed. The area around the wound is red and spreading from the original site. Symptoms of infected wounds are redness, swelling, pain, and pus. Second, neuropathic ulcers are the most common complication of uncontrolled diabetes. The wound is usually round in shape, with a deep hole in the middle part and thickened skin on the edges. It is found at the base of the big toe and pinky toe. Neuropathic ulcers develop from smaller wounds with symptoms of redness, swelling, numbness, or inflammation around the affected area. Third, ischemic ulcers are wounds that develop when the arteries do not deliver enough blood flow to a specific area, causing local ischemia in the skin and the underlying tissues. Initially, these ulcers manifest as blue discoloration, necrosis, or gangrene. They are painful wounds often found on the tips of the toes, especially the big and pinky toes. The characteristics of the wounds are pale, yellow, and cold, with a weak or absent pulse. They often start at the fingertips and pose an increased risk of amputation if left untreated. Diabetic ulcers are complex and chronic wounds that cannot follow the normal wound-healing process. When the wound is infected, dead tissue (sloughing or shedding) and purulent exudate from inflammation of the wound. There is no new tissue formation, and it does not heal within 4 weeks. Diabetic wounds heal slowly, and sometimes they do not heal. If the wounds are left without proper care, they will cause an infection that will spread to the bone layer (osteomyelitis), requiring organ removal (amputation). If a severe infection enters the bloodstream (septicemia), it can cause death. The essential goals in caring for patients with diabetic ulcers are to prevent disability from the wound spreading to the point where it is necessary to amputate the involved organ, to prevent new wounds from forming after treatment, and to increase the quality of life of the patients.

Diabetic wound care is integral to medical practice to heal wounds better and faster. To promote wound healing faster, (1) clean the wound to prevent infection and promote the wound healing process, (2) keep the wound elevated to reduce fluid accumulation and promote blood circulation, (3) provide adequate knowledge regarding nutrition to promote the wound healing process, (4) have enough rest because resting will reduce the metabolic process inside unnecessary cells and tissues and can carry enough oxygen and nutrients to the wounds, and (5) promote personal hygiene [6]. Diabetic wound care should be supervised by a nurse with clinical expertise who can collect, evaluate, analyze, and identify the needs and problems of patients. Expert nurses should be able to manage options for care and use medical supplies and equipment correctly and appropriately, obtain communication skills to coordinate with the treatment team and encourage family members to participate in care, identify health services and resources, protect patients’ rights and privacy, and preserve patient safety and prevent harm during care [7-9]

Despite a community point prevalence of approximately 1.5% to 5% for diabetic foot ulcers, the lifetime incidence risk reaches 19% to 34% [1]. This elevation is frequently linked to suboptimal foot self-care practices; however, there are not enough nurses to meet the increasing demand for care and other issues in Mae Chan District, Chiang Rai Province, such as a lack of health equipment; resources; and access to health services, communication channels for health care advice, and continuity of care rehabilitation. Therefore, strategies for caring for diabetic wounds are necessary, such as creating a network of channels for giving advice from specialists to nurses, caregivers, and patients in the community, monitoring and following up with patients, and providing quality of service through online channels. Various health care advice channels could increase access to services, reduce waiting time, and facilitate patients in a new way of life (New Normal). Moreover, the use of digital technology systems to provide health services for continuous care and referral to primary care units with the best practice guidelines, The Nursing and Midwifery Council has announced telenursing strategies to provide health care advice and to find solutions related to health within the knowledge framework of the nursing and midwifery profession [10].

Telenursing uses information and communication technologies to develop all fields of nursing care, education, and research at a distance [11]. In Thailand, the Nursing and Midwifery Council provides advice and solutions related to health within the knowledge framework by using this digital technology system to provide services and support all forms of nursing care, including continuous care, quality of care, equal access to health, and allowing people to manage their health through online services [10]. Telenursing care increases channel access to health services, covers health care in remote areas, especially in areas with travel restrictions, and maintains professional standards and quality. Patients also do not need to commute to the hospital, which helps reduce the cost of hospital admission and other related expenses while still allowing monitoring and following up on patients’ conditions through online channels.

The Mae Chan Hospital is located in the Chiang Rai Province. It is a middle-level hospital that can host up to 120 beds and support referrals from community hospitals in the joint service network of 4 districts: Mae Chan District, Mae Fah Luang District, Doi Luang District, and Chiang Saen District. The Mae Chan Hospital is responsible for caring for patients referred from community hospitals when the capacity of those hospitals in the 4 districts reaches the limit. The hospital helps increase access to treatment for service users in the area, reduces referrals to regional hospitals (advanced-level hospital), and supports primary service units in each district. The Wound and Ostomy Care Clinic, Mae Chan Hospital, provides services for patients with chronic wounds, bedsores, general wounds, penetrating wounds, drains, and ostomy care. Some nurses specialize in diabetic wounds, one specialist in ostomy care, and one in general care for patients. There were a number of people who received services from 2020 to 2022, including a total of 2142, 1571, and 1675 patients, representing 3725, 4931, and 6239 visits, respectively. There were 114, 70, and 67 patients with diabetic ulcers, accounting for 405, 281, and 291 visits, respectively. The average cost of commuting to the hospital for patients to receive wound care is US $10 per time. The average number of days to receive wound care services is 14 to 45 days per person. In general, the travel distance for patients to visit the wound care and ostomy clinic is 1 to 40 km. Due to travel distance and expenses involved in wound care at the hospital, some of the patients are unable to receive treatment regularly and continuously, so many patients experience wound spreading and recurrence of wound infection, leading to foot or leg amputation in some cases.

According to literature reviews, telenursing is important in assisting patients to access standard care for chronic wounds. Telenursing care helps reduce complications and the number of patients admitted to the hospital, which encourages participation among patients, caregivers, and nurses in self-care practices, and increases patient satisfaction because it reduces travel time, traveling costs, and other related expenses of going to the hospital. Therefore, the research team was interested in studying the development of telenursing guidelines for caring for patients with diabetic wounds and foot ulcers, and the effects of telenursing on wound healing in patients to provide quality service and prevent the spread and recurrence of wound infections and foot and leg amputations in patients.

## Methods

### Study Design

This was participatory action research [12], which divided the study into 3 cycles.

#### Cycle 1

The current situations, problems, and needs of patients with diabetic wounds and the feasibility of using telenursing guidelines in the context of the Mae Chan Hospital, Chiang Rai Province, and community networks in the research area were studied. The researcher used lessons learned from the study to conclude the issues and needs of developing and drafting telenursing guidelines for diabetic wound care. Informants and participants in the design of diabetic wound care guidelines and telenursing system were purposively selected, consisting of (1) patients with diabetic wounds and/or their caregivers who received care in the Wound and Ostomy Care Clinic, totaling 10 persons; (2) the multidisciplinary team including a surgeon, a pharmacist, a nutritionist, and 9 registered nurses at subdistrict health-promoting hospital in the Mae Chan Hospital networks, totaling 12 people; and (3) a computer technical officer.

The interprofessional team plays a critical role in providing comprehensive care for patients with chronic diabetic wounds. The surgeon is central to diagnosing the underlying causes of chronic wounds, determining their etiology, and formulating an integrated treatment plan. In collaboration with the health care team, the surgeon ensures the effective implementation of telenursing guidelines, addressing the needs of patients who face barriers to in-person health care access. The pharmacist works closely with the physician and nursing staff to select appropriate topical treatments that promote wound healing and minimize the risk of infection. In addition to this, the pharmacist is responsible for educating both patients and their families on the proper use of medications and wound care techniques. The nutritionist plays a vital role by recommending dietary strategies that support wound healing, promote tissue regeneration, and address underlying risk factors such as poor circulation or diabetes. Their tailored nutritional guidance is designed to strengthen the immune system and accelerate recovery. Finally, the computer technical officer ensures the seamless operation of the telemedicine system, facilitating smooth communication via platforms like the “Wound Care Mae Chan” LINE app. They provide technical support, enabling real-time updates and enhancing the overall telehealth experience for both health care providers and patients.

#### Cycle 2

Telenursing guidelines toward diabetic wound healing were studied. Participants recruited in the study were patients with diabetic wounds and professional nurses from subdistrict health-promoting hospitals under the Mae Chan Hospital network, totaling 20 persons. A sample size calculation was done using G*Power software version 3.1.9.4 (Fual et al), with test power set at 0.70, confidence value at 0.05, and influence value (effect size) at 0.30. The number of samples obtained from the calculation was 13. An additional 50% was added to the sample size, so the total number of participants in this cycle was 20.

#### Cycle 3

The effects of telenursing on diabetic wound healing were studied by evaluating the wounds, foot or leg amputation, and satisfaction with using telenursing guidelines. A quasi-experimental one-group pretest-posttest design was employed in this stage of the study.

### Research Instruments

The tools used for data collection are given below:

1. General information on characteristics was collected, including gender, age, education level, occupation, history of smoking, history of diabetes and duration, and types of diabetic wounds.
2. Bates-Jensen Wound Assessment Tool (BWAT): the BWAT consists of 13 wound characteristics: size, visible depth, wound edges, undermining and tunneling processes, necrotic tissue type and amount, exudate type and amount, surrounding skin discoloration, peripheral tissue edema, peripheral tissue induration, granulation tissue, and epithelialization [13]. The scores range from 13 to 65, and content validity and concurrent validity are both equal to 0.91. The assessment tool was used in a tryout among 10 patients with similar characteristics to find reliability using the Cronbach α coefficient formula, and the reliability value was 0.88. The cutoff scores of BWAT are the following: minimal scores are 13 to 20, mild severity scores are 21 to 30, moderate severity scores are 31 to 40, and critical severity scores are 41 to 65.
3. The questionnaire on satisfaction with the telenursing guidelines consists of 7 items rated on a 5-point Likert scale measuring satisfaction, with response options ranking from 5 (very satisfied), 4 (satisfied), 3 (neutral), 2 (dissatisfied), and 1 (very dissatisfied).

The tools used to conduct the study included the following:

1. Guidelines for diabetic wound care: the researcher developed the guidelines based on the existing literature review by specifying keywords to search for papers and related documents, including diabetic wounds, chronic wounds, guidelines for wound care, and wound care. The literature review search period was set from 2014 to 2023. Information was also obtained from cycle 1, which involved studying the current situations, problems, and needs of patients with diabetic wounds and the feasibility of using telenursing guidelines. The researcher presented a draft of telenursing guidelines to 3 experts in the fields of surgery, preventive medicine, and wound and ostomy care. These experts reviewed the guidelines to ensure they were consistent with the research objectives. Content validity was performed by measuring the Index of Item Objective Congruence, which was equal to 0.80.
2. LINE app “Wound Care Mae Chan”
3. Telemedicine Service System (Telemedicine; ITELE) of Mae Chan Hospital

### Ethical Considerations

The Human Research Ethics Committee at Chiang Rai Provincial Public Health Office approved this research study under approval 37/2023, with certification granted on April 1, 2023. The researcher adhered strictly to ethical principles throughout the study. Before participation, the researcher clearly communicated the objectives of the study, the research process, the expected duration, and the anticipated benefits to ensure a full understanding; subsequently, written informed consent was obtained from all participants before any data collection commenced. Informants and participants were informed that communication regarding wound care would be conducted via the LINE application, specifically designated for this research group. They were also informed of their right to withdraw from the study at any time, without any impact on their current treatment or benefits received from the hospital.

Participants received a remuneration of US $6 for each completed data collection session. The researchers were committed to protecting the participants’ rights and privacy. As such, all data were recorded using codes instead of real names, ensuring anonymity. All participant information was kept confidential and could not be traced back to any individual. The findings of the study were presented as aggregated data to protect participant identity. Furthermore, all recorded data, including images, would be destroyed 1 year after the publication of the research results.

## Results

As per the study objectives, we developed telenursing guidelines for caring for patients with diabetic wounds and foot ulcers, and studied the effects of telenursing on wound healing, using participatory action research.

In cycle 1, to study the current situations, problems, and needs of patients with diabetic wounds and the feasibility of using telenursing guidelines, we recruited 10 informants who were patients with diabetic wounds and foot ulcers aged between 30 and 74 years (average age 62.6, SD 12.4 years; duration of diabetes 1-10 years; average duration of diabetes 4.9, SD 3.5 years; average wound assessment score 51.3, SD 3.3); additional informants included a surgeon, a pharmacist, a nutritionist, 9 registered nurses at the subdistrict health-promoting hospital, and a computer technical officer. The following needs and problems were found.

Some patients could not receive wound care regularly and continuously because of the distance to the hospital, traveling difficulties, expenses for transportation, food, medical care, and related costs. Findings showed that some patients live far from the hospital. It costs them a lot of money per hospital visit. In some cases, patients have no one to take them to the hospital because family members have to work, and if they have to accompany the patients to the hospital, they have to miss their work and lose income. Moreover, they must spend extra money on transportation and food while waiting for patients to receive wound care. The cost of traveling to the hospital to receive wound care services is approximately 100 to 1000 Baht (US $2.85-$28.50) per visit, which is too much for them to spend, especially for patients and their family members who live in a remote community area.

Lack of knowledge about diabetic wound care is one of the problems. Patients lack adequate knowledge of wound care, nutrition, and control of blood sugar levels. These are factors for wound healing. In some cases, patients did not follow up on the appointment, which could also affect their physical health. Because of this, many patients experienced wound spreading and recurrence of wound infection, and in some cases, may experience foot or leg amputation.

Treating severely infected wounds requires skills and expertise in advanced wound care, which exceeds the potential of community nurses working in subdistrict health-promoting hospitals, and patients did not have access to or resources for advanced wound care, such as hydrocolloid, hydro fiber, alginate, hydrogel, foam, and film dressings.

**Figure 1.**
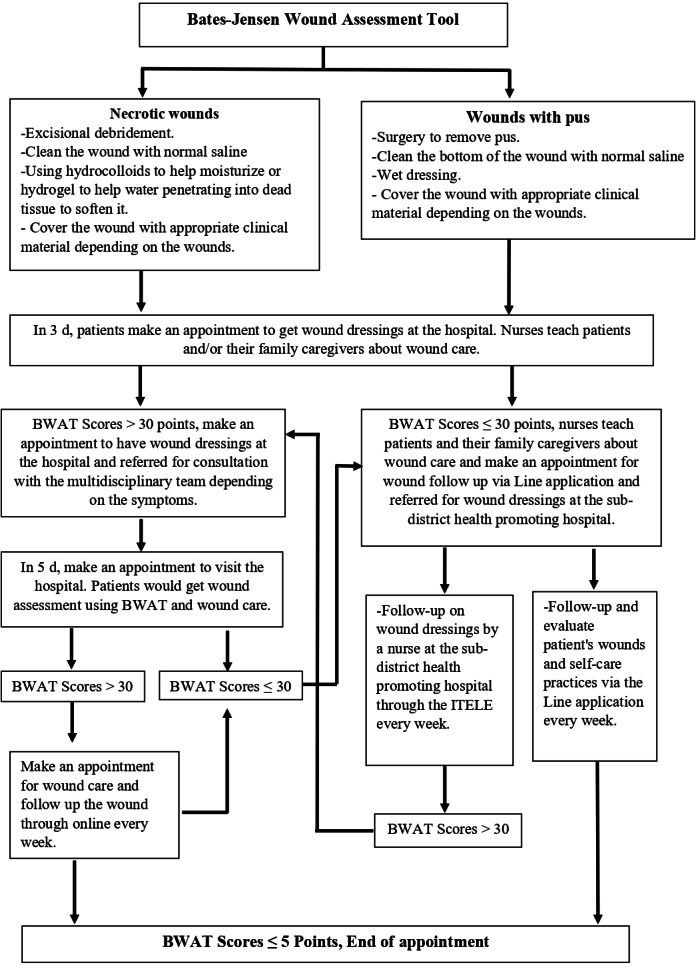
Telenursing guidelines for diabetic wound care. BWAT: Bates-Jensen Wound Assessment Tool; NSS: normal saline solution.

Nowadays, the majority of patients and their family caregivers have smartphones that can be used to communicate via the LINE app. Introducing telenursing for diabetic wound care among patients could help reduce barriers to receiving health care services. Based on the study of situations, needs, and problems among patients with diabetic wounds, telenursing guidelines for diabetic wound care are summarized as shown in Figure 1.

In cycle 2, the telenursing guidelines for diabetic wound healing were evaluated.

The study sample included 20 patients with diabetic wounds and foot ulcers, and half of them were female (n=10, 50%) and aged 30 to 81 years, with an average age of 47.6 (SD 9.4) years and an average duration of diabetes of 7.6 (SD 6.4) years. Studies showed that the wounds were found most on the leg, foot, and toe (n=15, 75%). The average assessment wound severity score was 50.8 (SD 4.4), indicating that patients had wound critical severity (BWAT=41‐65 points represents critical severity), and the results are shown in Table 1.

**Table 1.** Attributes of the sample.

Items	Value
Sex, n (%)	
Male	10 (50)
Female	10 (50)
Age (y)	
30‐49, n (%)	2 (10)
50‐59, n (%)	3 (15)
60‐69, n (%)	9 (45)
≥70, n (%)	6 (30)
Mean (SD)	62.6 (12.4)
Duration of diabetes (y), n (%)	
0‐2	4 (20)
3‐5	6 (30)
6‐10	7 (35)
≥10	3 (15)
Wound location, n (%)	
Head and neck	1 (5)
Arm, hand, and fingers	4 (20)
Legs, feet, and toes	15 (75)
Pain, n (%)	
Pain occurs during wound dressing	7 (35)
Pain is present at all times	8 (40)
Intermittent pain	5 (25)
Wound status score	
Mean (SD)	50.8 (4.35)
Critical severity (BWAT^a^=41‐65 points), n (%)	20 (100)
Wound characteristics, n (%)	
Size (length × width) (cm^2^)	
<4	3 (15)
4-16	11 (55)
16.1-36	2 (10)
36.1-80	3 (15)
>80	1 (5)
Depth	
Obscured by necrosis	5 (25)
Full-thickness skin loss with extensive destruction, tissue necrosis, or damage to muscle, bone, or supporting structures	15 (75)
Wound edges	
Indistinct, diffuse, and none clearly visible	19 (95)
Distinct, outline clearly visible, attached, and even with wound base	1 (5)
Undermining	
<2 cm in any area	14 (70)
2‐4 cm involving <50% wound margins	6 (30)
Necrotic tissue type	
Loosely adherent yellow slough	3 (15)
Adherent, soft, and black eschar	17 (85)
Necrotic tissue amount	
>50% and <75% of wound covered	14 (70)
75%-100% of wound covered	6 (30)
Exudate type	
Serosanguineous: thin, watery, and pale red or pink	1 (5)
Serous: thin, watery, and clear	7 (35)
Purulent: thin or thick, opaque, tan or yellow, and with or without odor	12 (60)
Exudate amount	
The amount of secretion is ≤25%	1 (5)
The amount of secretion is more than 25%‐75%	7 (35)
The amount of secretion is more than 75%	12 (60)
Skin color surrounding the wound	
Bright red and/or blanches to touch	16 (80)
Black or hyperpigmented	4 (20)
Peripheral tissue edema	
Pitting edema extends <4 cm around the wound	9 (45)
Crepitus and/or pitting edema extends >4 cm around the wound	11 (55)
Peripheral tissue induration	
None present	1 (5)
Induration, <2 cm around the wound	2 (10)
Induration 2‐4 cm extending <50% around the wound	6 (30)
Induration 2‐4 cm extending >50% around the wound	11 (55)
Granulation tissue, n (%)	
Bright, beefy red; <75% and >25% of wound filled	3 (15)
Pink and/or dull, dusky red and/or fills <25% of wound	17 (85)
Epithelialization, n (%)	
50% to <50% wound covered	3 (15)
<25% wound covered	17 (85)

a BWAT: Bates-Jensen Wound Assessment Tool.

The researcher prepared workshops and set up a meeting to explain the task of developing telenursing guidelines to the committee and those responsible for the continuous care, advanced wound care, and training program using the ITELE.

In cycle 3, mean wound severity scores were compared before and after receiving telenursing guidelines for diabetic wound care at week 2, week 4, week 6, and week 8 using 1-way repeated measures (ANOVA) statistical analysis; the results are shown in Table 2. The mean scores for wound severity were significantly different at the .001 level. A comparison of the wound severity scores before and after receiving telenursing nursing care guidelines for wound care at week 2, week 4, week 6, and week 8 using *t* test (1-tailed) statistical analysis is shown in Table 3.

**Table 2.** Comparison of the wound severity scores before and after receiving telenursing guidelines toward diabetic wound care using repeated measures of 1-way ANOVA statistics.

ANOVA	Sum of square	*df*	Mean square	*F* test	*df*	*P* value
Between-group	15,379.4	4	3844.8	401.7	4	<.001
Within-group	3360.2	95	35.4	—^a^	95	—
Total	18,739.6	99	—	—	99	—

a Not applicable.

**Table 3.** Comparison of the wound severity scores before and after receiving telenursing guidelines towards diabetic wound care at week 2, week 4, week 6, and week 8.

	Wound severity scores, mean (SD)	*t* test (*df*)	*P* value
Before and after receiving telenursing care guidelines for diabetic wound care in the second week	23.7 (5.3)	20.0 (19)	<.001
Before and after receiving telenursing care guidelines for diabetic wound care in the fourth week	29.6 (3.9)	34.3 (19)	<.001
Before and after receiving telenursing care guidelines for diabetic wound care in the sixth week	32.0 (4.0)	35.3 (19)	<.001
Before and after receiving telenursing care guidelines for diabetic wound care in the eighth week	34.0 (4.7)	36.1 (19)	<.001
After receiving telenursing care guidelines for diabetic wound care in the week 2 and week 4	5.9 (3.9)	6.8 (19)	<.001
After receiving telenursing care guidelines for diabetic wound care in the week 2 and week 6	8.3 (5.0)	7.4 (19)	<.001
After receiving telenursing care guidelines for diabetic wound care in the week 2 and week 8	10.3 (6.5)	7.1 (19)	<.001
After receiving telenursing care guidelines for diabetic wound care in the week 4 and week 6	2.4 (2.6)	4.1 (19)	<.010
After receiving telenursing care guidelines for diabetic wound care in the week 4 and week 8	4.4 (3.9)	5.1 (19)	<.001
After receiving telenursing care guidelines for diabetic wound care in the week 6 and week 8	2.0 (2.3)	3.8 (19)	<.010

According to Table 3, the wound severity scores after receiving telenursing care in the 2nd, 4th, 6th, and 8th weeks had decreased statistically (*P*<.001). The scores after receiving telenursing care in weeks 4, 6, and 8 had decreased statistically from week 2 (*P*<.001). The scores after receiving telenursing care in weeks 6 and 8 had decreased statistically from week 4 (*P*<.001), and the scores after receiving telenursing care in week 8 had decreased statistically from week 6 (*P*<.001).

## Discussion

### Principal Findings

The findings of this study showed that samples had a mean age of 62.6 (SD 12.4) years, an average duration of having diabetes for 4.9 (SD 3.5) years, and high levels of wound severity on average (BWAT=50.8, SD 4.3). This indicates that the majority of the sample were older people, who have a high risk for critical wound severity. First, the factors affecting the wound healing process include infection, high blood sugar levels, and nervous system disorders. This is consistent with the study by Burgess et al [14], which found that the pathophysiology related to the diabetic wound healing process includes hyperglycemia, neuropathy, microvascular complications, infection, inflammation, and immune system deficiency in chronic wounds, and the psychological impacts of diabetes mellitus. Second, the factors preventing patients from receiving wound care continuously include financial limitations, distance to the hospital, no caregivers taking them to the hospital, and so on. From the in-depth interviews, it was found that the majority of patients live far from the hospital. Distance is one of the barriers to accessing health services. Some patients do not have anyone to care for because family members have to work. Due to this reason, some of the patients are unable to go to the hospital by themselves. If a caregiver comes to the hospital, it would cost more money for traveling expenses, which is more than the net income received each day. The average cost is US $6 to US $10 per time, which is considered a high expense. Third, the knowledge factors where the patient does not have enough knowledge regarding blood sugar control and nutrition, which are important factors to promote wound healing. The problems and needs of patients led to the development of telenursing guidelines to provide patients with access to wound care services to prevent leg or foot amputation, increase the rate of wound healing, reduce wound healing time, reduce inequality, and distribute opportunities to access advanced wound care and resources for wound healing.

A study of the development of using telenursing guidelines on diabetic wound healing found that the mean wound-based severity scores had decreased significantly after receiving telenursing guidelines for diabetic wounds in week 2, week 4, week 6, and week 8 (*P*<.001). Moreover, no patients were found to have foot or leg amputation. This can be explained by the fact that the samples in this study received baseline assessment, including illness history, duration of the wound, history of received medicine, financial status, nutrition, and wound assessment using the BWAT. Patients also received wound assessment every week from the beginning throughout the study. Having regular wound assessments is important for effective wound care. Understanding factors affecting wound healing helps nurses make appropriate decisions in wound care [15]. This allows the wound healing process to proceed normally, and it works well together by preparing the wound to be in an appropriate state according to the principles of wound bed preparation [16]. Wound management consists of three phases. First, the debridement of the wound is when dead or unhealthy tissue is removed from a wound. It stimulates wound healing to the proliferative phase, causing re-epithelialization of the wound to be smaller, which prevents or reduces the amount of infection by removing pathogenic parts such as foreign bodies. Second, the wounds are made moist by dealing with secretions, making the wound healing more effective and causing angiogenesis, the formation of granulation tissue, and the process of re-epithelialization. Third, the wound infection is controlled by cleaning the wound to reduce the number of germs and giving topical antibiotics [17,18].

Information for developing telenursing guidelines was obtained from studying the situations, problems, and needs of patients with diabetic wounds and foot ulcers, as well as from reviewing the existing literature. Based on the information, self-care behaviors are important for promoting wound healing among patients, such as controlling blood sugar levels, taking care of the wound by cleaning and getting wound care regularly and continuously, and eating proper food with good nutrition. According to this study, the patients in the group that received telenursing care could heal wounds in an average of 8.6 (SD 4.3) weeks. The patients in the group that received advanced wound care and normal wound care could heal their wounds in an average of 10.2 weeks. The patients in the group that received wound care every day (conventional wound dressings) and normal wound care could heal in an average of 11.72 weeks. The average cost of wound care for the group receiving telenursing guidelines was 11,772 Baht (US $336.34) per person, receiving advanced wound care and normal care was 14,772 Baht (US $422.06) per person, and receiving wound care and normal care every day was 22,740 Baht (US $649.71) Baht per person. In terms of the average score of satisfaction using telenursing guidelines, it was 4.7 out of 5 (SD 0.2). This is consistent with many studies stating that self-care behaviors, blood sugar level control, and continuous use of medication among patients with diabetes were better after telenursing care [19-22], and this reduces the use of resources for treating diabetic wounds, hospital admissions, and patient expenses while maintaining service quality [19,23-26].

This research is in alignment with both national and international policies that promote the incorporation of digital technology as a tool for enhancing health care services. It also functions as a conduit, bridging the gap between communities and health care systems, thereby mitigating geographical and relational disparities between the public and health care providers. Moreover, digital technology empowers individuals to stay informed about evolving health conditions, understand their rights to access high-quality, equitable, and safe health care, and fosters public awareness and collaboration in improving personal and community health outcomes [27,28].

### Suggestions for Using Research Results

To continuously produce good results when implementing telenursing guidelines for improving the quality of services in diabetic wound care, those involved in the development process, including community people, patients, health care providers, nurses, health staff, and family caregivers, must be prepared in terms of knowledge in the field of diabetic wound care such as advanced wound care, the ITELE program, community nursing, self-care practices and knowledge regarding the use of remote technology in accordance with the steps of the nursing process, and health assessment, including defining problems, planning, practice, and evaluation.

There must be supervision, monitoring, and evaluation of the use of telenursing guidelines to improve nursing practices suitable to the community context and in accordance with knowledge about advanced wound care, diabetic wounds, chronic wounds, and remote technology.

### Limitations

The small sample size limits the ability to generalize the findings to a broader population.

## Abbreviations

BWAT: Bates-Jensen Wound Assessment Tool
